# Accessing the impacts of bamboo expansion on NPP and N cycling in evergreen broadleaved forest in subtropical China

**DOI:** 10.1038/srep40383

**Published:** 2017-01-09

**Authors:** Qing-ni Song, Hui Lu, Jun Liu, Jun Yang, Guang-yao Yang, Qing-pei Yang

**Affiliations:** 1 Ministry of Education Key Laboratory for Earth System Modeling, Department of Earth System Science, Tsinghua University, Beijing 100084, China; 2 The Joint Center for Global Change Studies, Beijing 100875, China; 3 Stable Isotope Center, College of Forestry, Fujian Agriculture and Forestry University, Fuzhou 350002, China; 4 Jiangxi Provincial Key Laboratory for Bamboo Germplasm Resources and Utilization, Jiangxi Agricultural University, Nanchang 330045, China

## Abstract

Bamboo (*Phyllostachys pubescens*) expansion into adjacent forests is a widespread phenomenon in subtropical regions, and it has greatly changed the dominance hierarchy from trees to bamboos. This process may be accompanied by changes in productivity, nutrients accumulation and biogeochemical cycles. We compared the net primary production (NPP) and major pools and fluxes of nitrogen (N) in bamboo-dominant forest (BDF) and neighboring secondary evergreen broadleaved forest (EBF) in South China using the space-for-time substitution method. We found that the mean NPP of the BDF was 30.0 t ha^−1^ yr^−1^, which was 51.5% greater than that of the EBF (19.8 t ha^−1^ yr^−1^). The plant N pool for the BDF was 37.5% larger than that of the EBF, whereas the soil inorganic N pool significantly decreased by 31.2% with conversion of the EBF to BDF. Additionally, the ratio of N return to N uptake was 0.69 in the BDF and 0.88 in the EBF because of the lower litter N return of the BDF compared with that of the EBF. These results indicated that the expansion of *P. pubescens* significantly increased the NPP and plant N accumulation but reduced the soil N available pool and slowed the N cycling rate, which could lead to soil degradation.

Bamboos(Poaceae: Bambusoideae) are perennial tree-like grasses widely distributed in tropical, subtropical and temperate forest ecosystems^1^. These plants frequently expand into their neighboring forest ecosystems by clonal and sexual reproduction and become dominant species in the canopy or understory with human or natural disturbances^2^,^3^,^4^. Here, this phenomenon is referred to as “bamboo expansion”. Bamboo expansion may significantly impact the colonized forests with regards to the species composition^5^,^6^, community structure^2^,^7^, and forest landscapes^8^,^9^, all of which have drawn worldwide attention of ecologists.

Moso bamboo (*Phyllostachys pubescens* Mazel ex J. Houz.) is the most broadly distributed bamboo species in subtropical China^10^. Because it grows quickly and has a leptomorph (running) rhizome system, *P. pubescens* can spread rapidly into disturbed forests as well as undisturbed forests, including areas characterized by shrubs^11^, coniferous forest^12^, mixed needle and broadleaved forest^13^ and pure broadleaved forest^14^,^15^. The unbridled expansion of *P. pubescens* has imposed great ecological consequences on these forests^4^,^13^, therefore, it has been considered as one of the greatest contemporary threats to woody forests in China.

Evergreen broadleaved forest (hereafter, EBF), the typical vegetation type in subtropical areas, is recognized as an important global vegetation type that contributes to both biodiversity and environmental protection^16^. Despite their formerly widespread geographical distribution, remnant EBFs next to bamboo forest unfortunately are now suffering from extensive expansion by bamboo^15^,^17^. Until now, a viable strategy has not been proposed for preventing bamboo expansion into EBFs, even in natural reserves and ecological public-welfare forest areas. Therefore, the ecological effects of *P. pubescens* expansion on EBFs must be assessed.

Recent studies have reported the loss of plant diversity^13^ following *P. pubescens* expansion, and simplified community structure^2^ and reduced tree regeneration^8^ because bamboo are more competitive with regard to capturing light and other resources^18^,^19^. Changes in the species composition from trees to bamboos are expected to exert great influences on the net primary productivity (NPP) and biomass of evergreen broadleaved forest, because bamboo are characterized by active rhizomatous clonal growth and high belowground allocation of biomass^10^. For example, Yang *et al*.^20^ found that *Phyllostachys edulis* expansion affected biomass accumulation of evergreen broad-leaved forest in Dagangshan mountain. Apart from its effects on plant biomass and production, nitrogen dynamics are essentially driven by the development of a bamboo biomass pool. It is likely that bamboo have relatively higher leaf N concentration than those of the dominant species in broadleaved forest^14^. This shift in tissue quality, along with a shift toward greater belowground biomass, will alter aboveground N pools and soil N pool, as well as litter dynamics. Changes in nitrogen cycling would in turn affect the ecosystem NPP and biomass accumulation^21^,^22^. However, to date, little is known about the underlying N biogeochemistry cycling, which is crucial for better understanding of ecosystem functioning after expansion.

Based on the increasing awareness of bamboo expansion, pairs of bamboo–dominated forests (hereafter, BDF) and EBFs in Dagangshan National Forest Ecological Station in Jiangxi Province of China were used as the research objects. We aimed to comprehensively assess the ecological consequences of biomass, production, N pools and fluxes after the expansion of *P. pubescens* into adjacent EBFs. Our specific objectives are to (1) quantify the size and distribution of biomass and net primary production (NPP) and (2) compare the main ecosystem N pools and N fluxes in BDF and front uninvaded EBF growing on similar soils. Our results can provide experimental data and parameters to evaluate the ecological effects of *P. pubescens* expansion into native, broad-leaved forest in equivalent or similar climatic areas, which can help us better understand how bamboo expansion affects soil N pool and serve as a scientific basis for restoration of bamboo forest conversion to evergreen broadleaved forests.

## Results

### Shifts in vegetation biomass

**Figure 1:**
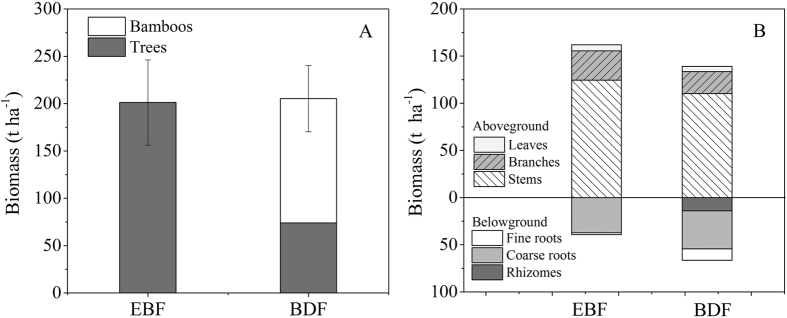
Allocation of biomass (t ha^−1^) in evergreen broadleaved forest (EBF) and bamboo-dominant forest (BDF) in Dagangshan National Forest Ecological Station, Jiangxi Province, China. (**A**) Species allocation; and (**B**) organ allocation. The data are expressed as the mean ± SD; SD is the standard deviation (n = 3).

Significant differences were generally not observed in total community biomass between the EBF and BDF (201.2 t ha^−1^ vs 205.5 t ha^−1^) (F_1, 4_ = 0.17, P = 0.579). It was because the decrease of the tree biomass (127.1 t ha^−1^) was offset by the increase of the bamboo biomass (131.2 t ha^−1^) from EBF to BDF (Fig. 1A). The biomass spatial allocation clearly varied after bamboo expansion. The aboveground biomass (W_A_) of the BDF (139.1 t ha^−1^) was 14.1% lower than that of the EBF (161.9 t ha^−1^) (F_1, 4_ = 35.65, P = 0.021), with the W_A_ of the stems, branches and leaves reduced by 11%, 25% and 14%, respectively. Conversely, the belowground biomass in the BDF was 66.4 t ha^−1^, which was 69.0% greater than that of the EBF (39.3 t ha^−1^) (F_1, 4_ = 40.49, P = 0.024). Consequently, the ratio of the aboveground biomass to belowground mass (W_A_/W_B_) significantly decreased from 4.1 in the EBF to 2.1 in the BDF (F_1, 4_ = 22.44, P = 0.042).

### Shifts in vegetation NPP

**Figure 2:**
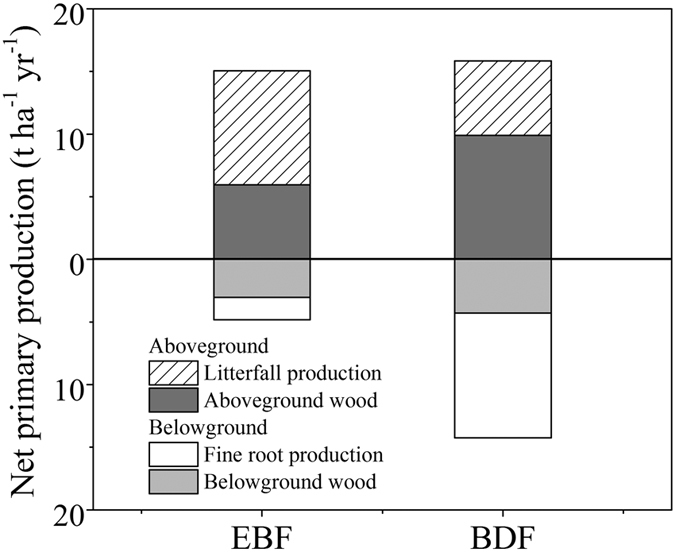
Allocation of net primary production (t ha^−1^ yr^−1^) of evergreen broadleaved forest (EBF) and bamboo-dominant forest (BDF). The data are expressed as the mean ± SD; SD is the standard deviation (n = 3).

Between 2007 and 2011, the mean annual NPP for EBF and BDF was 19.8 t ha^−1^ yr^−1^ and 30.0 t ha^−1^ yr^−1^, respectively (Fig. 2), therefore, bamboo expansion led to a NPP increase of 51.5% (F_1, 4_ = 39.89, *P* = 0.024). This result was mainly caused by the more than 8.2 t ha^−1^ yr^−1^ of fine root production in belowground NPP of BDF than that of EBF. No significant differences were observed in the aboveground NPP between EBF and BDF (15.0 t ha^−1^ yr^−1^ versus 15.8 t ha^−1^ yr^−1^) (F_1, 4_ = 1.018, *P* = 0.906). Thus, the proportion of belowground components in the total NPP increased from 24.2% to 47.3% with the conversion of EBF to BDF.

### Plant N content

Almost all organs (except branches) of bamboo presented greater N content compared with the corresponding organs of broadleaved trees (Table 1). The order of N content among old bamboo shoots (>1 yr) organs was leaves > rhizomes > branches > coarse roots > stems, and all trees measured showed similar patterns in different organs. The N content for the organs of young bamboo individuals (≤1 yr) was higher than that of old individuals (>1 yr), particularly for the stems, leaves and rhizomes (Table 1).

### Shifts in ecosystem N pools

#### Plant N pool

**Figure 3:**
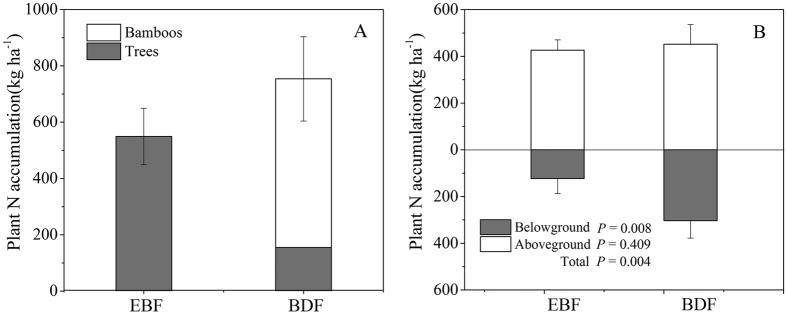
N allocation in the plant pool (kg N ha^−1^) of evergreen broadleaved forest (EBF) and bamboo-dominant forest (BDF). (**A**) Species allocation; and (**B**) organ allocation. The data are expressed as the mean ± SD; SD is the standard deviation (n = 3).

The plant N pool of the BDF was 756.0 kg N ha^−1^ (Fig. 3), which was 37.5% greater than that of EBF (550.0 kg N ha^−1^) (F_1, 4_ = 33.58, *P* = 0.004), and its allocation pattern of N was different in the two forests. The N accumulation in bamboos increased from 0 to 599.9 kg N ha^−1^, and that in trees decreased by 393.8 kg N ha^−1^ from the EBF to the BDF (Fig. 3A). The N pool of the aboveground compartments were 426.0 kg N ha^−1^ and 452.1 kg N ha^−1^ for the EBF and BDF, respectively (F_1, 4_ = 2.07, *P* = 0.109); however, the N pool of the belowground compartment shifted from a value of 124.0 kg N ha^−1^ for the EBF to 303.9 kg N ha^−1^ for the BDF (Fig. 3B), which represented an increase of 145.1% (F_1, 4_ = 122.56, *P* = 0.008).

#### Litter N pool

**Figure 4:**
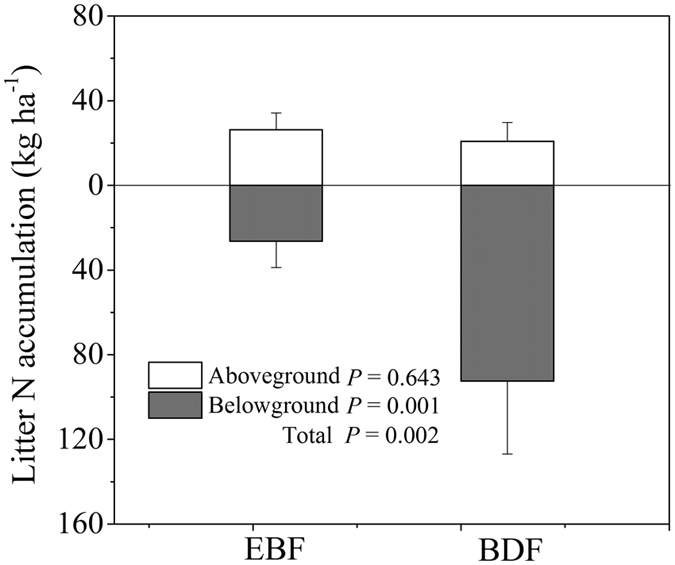
N allocation in the litter pool (kg N ha^−1^) of the evergreen broadleaved forest (EBF) and bamboo-dominant forest (BDF). The data are expressed as the mean ± SD; SD is the standard deviation (n = 3).

A significant increase in the total litter N pool was observed with the conversion of EBF to BDF (53.0 kg N ha^−1^ versus 113.0 kg N ha^−1^) (F_1, 4_ = 26.84, *P* = 0.002), with the belowground litter contributing the greatest amount for the BDF (Fig. 4). The litter N pool was 3.5-fold greater in the belowground litter of the BDF relative to that of the EBF (92.4 kg N ha^−1^ versus 26.4 kg N ha^−1^) (F_1, 4_ = 32.33, *P* = 0.001), whereas the aboveground litter N pool did not show differences between the BDF and EBF (20.8 kg N ha^−1^ and 26.3 kg N ha^−1^, respectively) (F_1, 4_ = 0.25, *P* = 0.643).

#### Soil N pool

**Figure 5:**
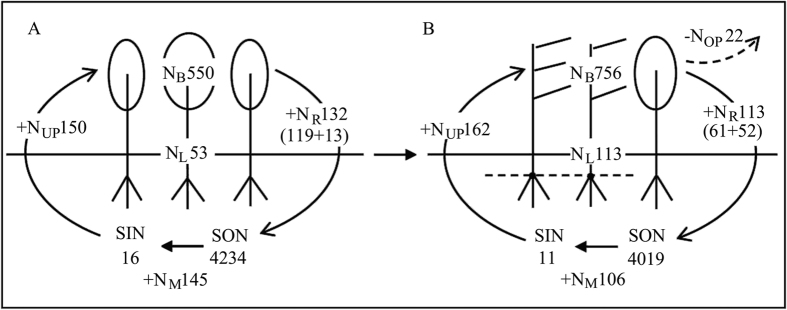
Changes in the distribution pattern of N pools (kg ha^−1^) and N fluxes (kg ha^−1^ yr^−1^) after bamboo expansion into neighboring evergreen broadleaved forest. (**A**) Evergreen broadleaved forest, EBF; and (**B**) bamboo-dominant forest, BDF. N pools included plant biomass (N_B_), standing litter (N_L_), soil organic N(SON) and inorganic N(SIN) within 0–20 cm. N fluxes included annual plant N uptake (+N_UP_), N return by litter (+N_R,_ the former data in the parenthesis is N return by aboveground litterfall, the latter is by fine root mortality)), net N mineralization rate (+N_M_), and N output from harvested bamboo (−N_OP_). Values are the mean of three plots from each stand.

The soil inorganic N (SIN) of BDF at a soil depth of 0–20 cm was 11.0 kg N ha^−1^ (Fig. 5), which was 31.2% lower than that of EBF (16.0 kg N ha^−1^) (F_1, 4_ = 8.25, *P* = 0.045), but the soil organic N (SON) pool did not show significant differences between EBF and BDF (4234 kg N ha^−1^ versus 4019 kg N ha^−1^) (F_1, 4_ = 0.85, *P* = 0.43).

### Shifts in ecosystem N fluxes

The annual N uptake by plants in the BDF (162.0 kg N ha^−1^ yr^−1^) was slightly higher than that in the EBF (150.0 kg N ha^−1^ yr^−1^) (F_1, 4_ = 4.19, *P* = 0.109); however, the total litter N return (aboveground + belowground) to soil was significantly lower in the BDF than the EBF (113.0 kg N ha^−1^ yr^−1^ versus 132 kg N ha^−1^ yr^−1^) (F_1, 4_ = 11.24, *P* = 0.041), thus representing a decrease of 14.4% (Fig. 5). The N cycling rate calculated as dividing the N return by N uptake was 0.69 in the BDF and 0.88 in the EBF, representing a 21.6% decrease for the BDF (F = 7.13, *P* = 0.056). Additionally, the N output from the BDF because of the harvesting of dead or old bamboos was approximately 22.0 kg N ha^−1^ yr^−1^; however, this output was neglected (Fig. 5).

## Discussion

### Expansion of bamboo changed vegetation biomass allocation pattern

Forest biomass is one of the best indicators of ecosystem function^23^. We found that the expansion of *P. pubescens* had few effects on the total vegetation biomass (Fig. 1). The averaged values of EBF biomass obtained in the present study was 201.2 t ha^−1^, a result that was higher than the mean value of EBFs in subtropical China (163.7 t ha^−1^)^23^ but close to the EBF in east China (225.3 t ha^−1^) reported by Yang *et al*.^24^. The total biomass of the BDF was 205.5 t ha^−1^, which was in the range of 132.8–309.2 t ha^−1^ previously reported for equivalent vegetation in various regions of China and Japan^14^,^25^,^26^. The biomass showed limited changes because the increase in bamboo biomass (131.2 t ha^−1^) was nearly sufficient to compensate for the loss of tree biomass (127.1 t ha^−1^) (Fig. 1A); although the vegetation density and BA increased approximately 5-fold and 1-fold after the *P. pubescens* expansion, respectively (Table 2), the biomass of hollow culm of individual bamboo was less than the biomass of individual tree in the same size. Our findings were consistent with those reported by Fukushima *et al*.^14^, who noted that an obvious trend of biomass did not occur in conjunction with temperate secondary broadleaved forest conversion into bamboo forest.

The biomass spatial distribution pattern was modified by the bamboo expansion. The ratio of the aboveground biomass to the belowground biomass (W_A_/W_B_) significantly decreased from 4.1 in the EBF to 2.1 in the BDF (Fig. 1B). This ratio is well within the range of 3–7 for broadleaved forest stands^23^ and 0.1–0.3 for grassland sites in China^27^. The stand structure of the BDF was between that of forest and grassland sites, and this structure is also suited for other bamboos, such as *Phyllostachys bambusoides* stands in Japan^28^ or *Guadua weberbaueri-*dominant open rain forests in Brazil^29^. A large amount of biomass allocated belowground in BDF. This is attributed to bamboos’ inherent extensive rhizome and root system^10^,^25^, which reflects a strategy for maintaining the advantageous regeneration of *P. pubescens* that could contribute to its rapid expansion^25^.

### Expansion of bamboo increased vegetation NPP

We found that the expansion of *P. pubescens* significantly increased the vegetation NPP. The NPP in the EBF estimated in the present study was 19.8 t ha^−1^ yr^−1^ (Fig. 2), which was close to the mean NPP value (14.5 t ha^−1^ yr^−1^) for subtropical EBFs^23^. The NPP of the BDF averaged 30.0 t ha^−1^ yr^−1^, which is equivalent to the 28.9 t ha^−1^ yr^−1^ of pure *P. pubescens* forest observed in the Wuyishan Biosphere Reserve of Southeast China^26^. This result was consistent with the findings of Isagi *et al*.^25^, who reported that *P. pubescens* forests had greater gross production compared with temperate broadleaved and/or coniferous forest stands in Japan. The greater total NPP after bamboo expansion was related to the greater belowground NPP, which was caused by the >8.2 t ha^−1^ yr^−1^ of fine root production in the BDF relative to that in the EBF (Fig. 2). Because fine roots are short lived and have a faster return^19^, their presence can explain the limited differences in biomass but greater NPP in the BDF compared with the EBF.

The NPP in forest ecosystems is often closely related to the forest type^30^, soil conditions^31^, and intrinsic plant properties^21^. Generally, higher NPP is found when soil N availability is higher (i.e., larger SIN pool and/or greater soil N mineralization rate)^31^,^32^,^33^. In this case, both the SIN pool and soil N mineralization rate were reduced after bamboo expansion (Fig. 5). Therefore, the difference in NPP between the two types of forest was likely independent of the soil N availability but related to the intrinsic advantages of the species themselves. Nitrogen use efficiency (NUE) of plant is important for governing the pattern of nutrient processes in many ecosystems^34^,^35^, and higher productivity could be promoted by higher NUE^21^,^35^. According to ecosystem-level NUE, which is calculated as the NPP per unit N returned in litter^34^, we found that the BDF has greater NUE (265.5) than the EBF (150.0). In addition, the leaf N concentration of *P. pubescens* exceeded the corresponding values of woody trees (Table 1) partly as a result of high leaf N reabsorption rate of bamboos^15^, indicating that *P. pubescens* has a higher photosynthetic ability^36^. Importantly, even *P. pubescens* in poor sites can obtain nutrients from rich sites by clonal integration to meet their N demand^37^. Therefore, more organic production can be generated in BDF with the increase of bamboo predominant. Regrettably, belowground components contribute most to the total NPP, leading to some aboveground functional shrinkage.

### Expansion of bamboo slowed ecosystem N cycling

N cycling in the ecosystem is primarily composed of three N pools (plant, litter and soil) and three N flux processes (N uptake, N return and N mineralization)^38^. The presence of *P. pubescens* interrupts N cycling in colonized ecosystems. The plant N pool and litter N pool increased remarkably under bamboo expansion. The values of the plant N pool were 550. kg ha^−1^ in the EBF and 756.0 kg ha^−1^ in the BDF (Fig. 3). The shift in the plant N pool was similar to that found in many studies of shrub invasion into grassland in which the accrual of plant N was promoted^35^,^39^,^40^. However, the shift was not consistent with the findings reported by Fukushima *et al*.^14^, who found that the plant N stock was not influenced by the expansion of *P. pubescens* into temperate broadleaved forest. The differentiation between two studies may be due to the invaded ecosystems between two studies were different in species composition, stem density, site conditions, and the interference degree by bamboos. The large plant N accumulation in the BDF in this study was caused by the higher N content in bamboo tissue than in trees (Table 1) because the biomass exhibited no changes between the two forests. The total litter N pool increased by 60.0 kg N ha^−1^ with the conversion of EBF to BDF (Fig. 4), mainly resulting from more than 66 kg N ha^−1^ belowground litter N accumulation in BDF. The belowground litter N accumulation generally depends on the N input through root mortality and N output into soil by decomposition^38^. Because the N input by the fine root mortality in BDF(52 kg ha^−1^ yr^−1^) was higher than that in EBF (13 kg ha^−1^ yr^−1^, Fig. 5) and fine root decomposition rates varied little between BDF and EBF^41^, more N was accumulated below ground in the BDF than EBF.

In contrast to the N pools for plant and litter, we found that the SIN pool significantly decreased from 16.0 kg N ha^−1^ to 11 kg N ha^−1^ after EBF conversion to BDF (Fig. 5), which indicated a depletion of the available N in the soil pool but only a slight decrease on the SON pool despite some SON was output through bamboo harvesting. A similar observation was found in areas colonized by dwarf bamboo (*Sasa kurilensis*) invasion, which caused a lower soil N available N in *Betula ermanii* forests in northern Japan^3^.

The size of the SIN pool was mainly determined by litter N release, soil N mineralization rate and plant N uptake^38^,^42^. The expansion of *P. pubescens* decreased the N return by litter and the soil N mineralization but increased the annual N uptake by plants. The annual N return by litter (aboveground + belowground) decreased, but the standing litter N accumulation increased with the conversion of EBF to BDF (Fig. 5), which suggested a slower N release rate (N return/N storage) from the litter to soil. In addition, the amount of soil N mineralization decreased from 145 kg N ha^−1^ yr^−1^ in the EBF to 106.0 kg N ha^−1^ yr^−1^ in BDF in the topsoil at 20 cm^15^ (Fig. 5), which represented a 26.9% reduction. The lower litter N release and soil N mineralization rate could be attributed to the changes of microorganism abundance, community structure and activities^17^,^43^,^44^. Studies found that the abundance of fungi were decreased after bamboo expansion into adjacent forests^44^, similar decreased pattern was found in the soil cellulose and xylanase activities^43^. The annual N uptake by the BDF (162.0 kg N ha^−1^ yr^−1^) was greater than that by the EBF (150.0 kg N ha^−1^ yr^−1^), indicating that the demand greatly exceeded the annual soil N supply by mineralization in the BDF. Thus, the lower litter N release rate, the lower N mineralization rate and the higher N uptake led to a smaller SIN pool after bamboo expansion.

Increase N accumulation in plant and litter intercepted the N flux from the aboveground to belowground, thereby slowing the N cycling in colonized ecosystem by bamboo expansion. Reduced N return and release, slower soil mineralization rate and increased N uptake by vegetation led to a small SIN pool when bamboo spread into the former broadleaved forests. A decrease in soil N availability and increase in N uptake resulted from bamboo–soil feedback loops, which could limit original tree growth, seed germination and future seedling establishment, thereby threatening plant biodiversity. However, the same factors would have fewer effects on bamboo growth and reproduction because of bamboo’s nutrient integration by extensive rhizomes^37^ and high N resorption rate^15^. These findings represent consequences of bamboo expansion and act as a mechanism for *P. pubescens* expansion, which implies that NPP and N cycling should be considered when remnant forests are conserved to prevent bamboo expansion and bamboo forests are restored to EBFs.

In summary, *P. pubescens* expansion can spatially modify biomass allocation by reducing aboveground and tree biomass although with a limited influence on the total vegetation biomass, and increase NPP (especially belowground) through higher NUE. In addition, bamboo expansion may hinder N cycling in the colonized ecosystems, as evidenced by higher N uptake, higher plant N accumulation, lower N return and lower soil N mineralization rate, which may lead to potential soil degradation. These findings have great implications for assessing the ecological consequences and understanding the mechanisms of *P. pubescens* expansion, and they also provide fruitful insights into cases of other bamboo expansions. However, the effects of *P. pubescens* expansions and proliferation into EBFs could vary over time as well as on a site-by-site basis. Therefore, the long-term dynamics of these expanding ecosystems should be investigated further to accurately monitor the changes in NPP and N cycling that accompany *P. pubescens* expansion and proliferation in a variety of forest systems.

## Methods

### Site description

This study was conducted at Dagangshan National Forest Ecological Station in Jiangxi Province, South China (27°30′–27°50′N, 114°30′–114°45′E). The region has a middle subtropical monsoon climate, an annual mean temperature of 15.8–17.7 °C, and extreme temperatures in January of −8.3 °C and July of 39.9 °C. The annual mean precipitation is 1591 mm, which mainly occurs from March to August. This region has a subgroup of mountain red and yellow soil with well-weathered coarse granite of the Presinian system^45^. The climax vegetation is EBF; however, *P. pubescens* is also a suitable species and is spreading into adjacent EBFs^20^.

To understand the impacts of bamboo expansion on ecosystem properties, a space-for-time substitution was used as the evaluation method^46^. In August 2011, three pairs of stands straddling BDF and unexpanded EBF were continuously selected. EBF was selected to represent a reference condition, and neighboring BDF was recognized as the experimental unit. A 20 m × 20 m plot was established in each stand with the same slope and aspect. The plots were considered to have similar historical community structures and soil conditions. Therefore, differences in the current NPP and N cycling were assumed to have resulted from the bamboo expansion. Details of this site’s soil physical and chemical properties have been previously described by Song *et al*.^15^.

The EBF was located in the front of the BDF, and it was approximately 50 years old and dominated by the evergreen broadleaved tree *Castanopsis fargesii*, accompanied by *Castanopsis sclerophylla, Symplocos laurina, Schima superba*, and *Symplocos sumuntia*. The trees’ density, height and basal areas were 1031 stems ha^−1^, 16.9 m and 22.3 m^2^ ha^−1^, respectively, of which *C. fargesii* contributed 58.0% of the total breast area (BA) (Table 2).

The BDF was formed when *P. pubescens* expanded into secondary EBF approximately 30 years ago. The BDF was dominated by *P. pubescens*, accompanied with some resident broadleaved trees, including *Symplocos laurina, C. fargesii* and *C. sclerophylla*. The stand density was 5379 stems ha^−1^, with a ratio of *P. pubescens* over broadleaved trees of 9:1 (by individual). The diameter at breast height (DBH) of the bamboo and trees was 9.8 cm and 16.2 cm, respectively, and the corresponding mean height was 12.9 cm and 11.7 cm, respectively. The BA was 46.2 m^2^ ha^−1^, with *P. pubescens* accounting for 81.0% (Table 2). Fertilization was not performed at the bamboo sites, and the old (>10 a) and dead bamboos were occasionally harvested for firewood or housing.

### Biomass and NPP estimation

#### Biomass

The diameter at breast height (DBH) and height (H) were recorded for all trees and bamboos with DBH >5 cm in all 6 plots in August, 2011. The ages of bamboos were also measured in each bamboo sites. The biomass of the stems, leaves, branches, and coarse roots (including stumps) of the species in each subplot was estimated from the DBH and H using the allometric equation *W* = a × (DBH^2^ × H)^^b^ (Supplementary Table S1), which was established with equivalent vegetation from the study site of Yang *et al*.^20^. To estimate the bamboo biomass of the belowground rhizome, 5 vertical blocks of 100 cm length, 100 cm width and 90 cm depth were dug in each bamboo plot. All of the bamboo rhizomes in the block were picked out, and separated into current-year and older-year components. These components were then dried at 80 °C for 72 h to determine the dry mass and N content. The biomass of the fine root (<2 mm) of BDF and EBF was obtained from Liu *et al*.^19^ in the same study site. The aboveground biomass (W_A_), belowground biomass (W_B_) and total biomass (W_T_) were calculated using the following formulas:


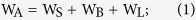



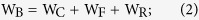



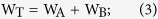


where W_S_, W_B_ and W_L_ represent the biomass of the stems, branches and leaves, respectively and W_C_, W_F_ and M_R_ represent the biomass of the coarse roots, fine roots and rhizomes, respectively.

#### NPP

The annual NPP, including the aboveground NPP (ANPP) and belowground NPP (BNPP) of vegetation were calculated using the following equations^47^:


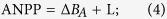



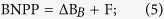



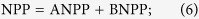


where *∆B*_*A*_ is the annual aboveground increment of wood biomass, including the stems and branches; *∆B*_*B*_ is the annual belowground increment of wood biomass, including the coarse roots and rhizomes; L is the annual aboveground litterfall production observed monthly from 2011 to 2012, obtained from Song *et al*.^15^; and F is the annual growth of fine root measured between 2010 and 2011, obtained from Liu *et al*.^19^.

The *∆B*_A_ and *∆B*_B_ of the trees were based on the DBH increment of each individual and calculated from a tree ring analysis. Based on the above biomass measurements, the species C. *fargesii, C. sclerophylla, S. laurina* and *S. superba* were selected from the plots using the average standard tree method (3 trees for each species) and cut down to analyze the age and annual ring width using the LINTAB™ 5 ring analyzer (Frank Rinn, Germany) of accuracy 0.01 mm. For each tree ring disc at DBH, an average incremental growth (in diameter) for the previous 5 years (2007–2011) was determined according to four directions. The mean annual incremental increase in DBH was then subtracted from the current diameter to estimate the average previous year’s diameter. To estimate the annual incremental increase of unmeasured species, we calculated the weighted mean increment based on the BA of each species in each plot. Previous year’s height was calculated by the relationship between DBH and H established by Yang *et al*.^20^ (Supplementary Table S1). Both the current DBH and H, the average previous year’s DBH and H were entered into the allometric equations to calculate biomass. The difference in biomass between the current and the previous year was the tree woody growth increment.

The annual production of *∆B*_A_ of living bamboo was calculated as the weight of new bamboo shoots. For the number of the new bamboo shoots has a regular biennial cycle, but was constant in each cycle^48^. Thus, we calculated the total aboveground biomass of living bamboos within recent 3 biennial cycles (2006–2011, 6 years). The *∆B*_A_ of living bamboo was calculated as dividing the total aboveground biomass by 6 according to Li *et al*.^48^. *∆B*_B_ of living bamboo is calculated from the ratio of aboveground woody biomass to the belowground biomass of bamboos. The average annual amount of harvested bamboo was calculated by the annual amount of the bamboo culm production.

### N pool and flux estimation

The stem, branch, leaf and coarse root of the main tree species were sampled to measure the N concentration in each stand. We selected three adult trees for each species in each plot. Four subsamples were selected randomly from each adult tree and mixed them together as one sample, thus there was a total of 9 samples for each species. A previous study showed that the N concentration of *P. pubescens* leaves varies significantly by culm age^49^. So, the aboveground compartments of *P. pubescens* were separately sampled on current-year shoots and those older than 1 year. All of the samples were transported to the laboratory, oven dried at 70 °C, and then ground and sieved through a 0.25-mm mesh to measure the N concentration. The fine root samples were obtained by the study published by Liu *et al*.^19^. The total N concentration was measured by the micro-Kjeldahl method, in which 0.25 g sample was digested in 5 ml concentrated H_2_SO_4_ with a catalyst mixture (CuSO_4_, K_2_SO4 and selenium powder) and then distilled^50^.

N pools were calculated using the following formulas:


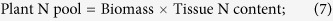



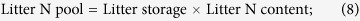



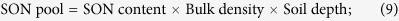



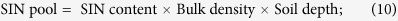


where the values of the standing litter storage, including aboveground compartments, were obtained from Song *et al*.^15^ and those for the belowground compartment (rhizome, coarse and fine root debris) were obtained from Liu *et al*.^41^. The SON content, SIN (NH_4_^+^-N+ NO_3_^−^-N) content and soil bulk density from the 0–20 soil layers were observed monthly between 2011 and 2012 from Song *et al*.^15^.

Main N fluxes were estimated using the following equations^47^:


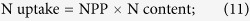



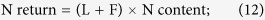



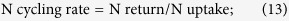



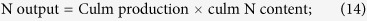


All of these estimates of standing storage for biomass and N are expressed as an oven-dry weight per hectare. N fluxes are expressed on an annual basis as the weight per hectare.

### Statistical analysis

An ANOVA was used to detect significant differences in the biomass, NPP, N storage, N uptake, N return and N cycling rate between the BDF and EBF. Differences in the N content of plant organs (including stems, branches, leaves, coarse roots, and rhizomes) between the bamboo and broadleaved tree species were detected by the ANOVA and a protected least significant difference test (LSD). *P* < 0.05 was the criterion for significant differences for all tests. Statistical analyses were performed using SPSS 17.0 for Windows.

## Additional Information

**How to cite this article:** Song, Q.-n. *et al*. Accessing the impacts of bamboo expansion on NPP and N cycling in evergreen broadleaved forest in subtropical China. *Sci. Rep.* **7**, 40383; doi: 10.1038/srep40383 (2017).

**Publisher's note:** Springer Nature remains neutral with regard to jurisdictional claims in published maps and institutional affiliations.

## Supplementary Material

Supplementary Table 1S

## Figures and Tables

**Table 1:** Mean N content of organs in bamboos and trees.

Species	Stems	Branches	Leaves	Coarse roots	Rhizomes
Bamboo shoot (≤1 a)	0.63 ± 0.19a	0.60 ± 0.16a	2.18 ± 0.35a	0.53 ± 0.16a	0.79 ± 0.11a
Bamboo shoot (>1 a)	0.22 ± 0.05b	0.47 ± 0.08a	1.75 ± 0.23b	0.42 ± 0.13a	0.51 ± 0.11b
*Castanopsis fargesii*	0.18 ± 0.02b	0.48 ± 0.06a	1.60 ± 0.96b	0.30 ± 0.12a	n.a.
*Castanopsis sclerophylla*	0.21 ± 0.05b	0.45 ± 0.12a	1.49 ± 1.20bc	0.34 ± 0.09a	n.a.
*Symplocos laurina*	0.13 ± 0.04b	0.55 ± 0.21a	1.43 ± 1.03bc	0.40 ± 0.12a	n.a.
*Schima superba*	0.16 ± 0.05b	0.56 ± 0.19a	1.01 ± 1.01c	0.38 ± 0.11a	n.a.

The data are expressed as the mean ± SD; SD is the standard deviation (n = 9). Lowercase letters in the same column indicate significant differences among different species in each stand at *P* = 0.05. n.a., not available.

**Table 2:** Structural characteristics of the secondary evergreen broadleaved forest (EBF) and bamboo-dominant forest (BDF).

	EBF	BDF
Stem density (stem ha^−1^)	1031 ± 82	529 ± 30
Culm density (stem ha^−1^)	n.a.	4850 ± 150
Trees DBH (cm)	16.9 ± 0.9	16.2 ± 1.9
Bamboo DBH (cm)	n.a.	9.8 ± 0.3
Tree height (m)	13.1 ± 3.8	11.7 ± 2.5
Bamboo height (m)	n.a.	12.9 ± 0.8
Total BA (m^2^ ha^−1^)	22.3 ± 2.7	46.2 ± 4.3
Number of species	12	11
Dominant species^+^	*Castanopsis fargesii* (58)	*Phyllostachys pubescens* (81)
Accompanying species^+^	*Castanopsis sclerophylla* (16)	*Symplocos laurina* (5)
	*Symplocos laurina* (12)	*C. fargesii* (3)
	*Schima superba* (6)	*C. sclerophylla* (2)

The data are expressed as the mean ± SD; SD is the standard deviation (n = 3). DBH is the diameter at breast height. BA is the stand breast area. ^+^The values in parentheses are the relative basal area of the species in the total BA (%). n.a., not available.
