# Supplementary material for: Accessing the impacts of bamboo expansion on NPP and N cycling in evergreen broadleaved forest in subtropical China
Source: Sci Rep. 2017 Jan 9;7:40383. doi: 10.1038/srep40383 (PMC5220298; doi:10.1038/srep40383)
Supplement: Supplementary Table 1S [file srep40383-s1.pdf]

Accessing the impacts of bamboo expansion on NPP and N cycling in evergreen broadleaved forest in subtropical China

Qing-ni Song[1], Hui Lu[1, 2, *], Jun Liu[3], Jun Yang[1, 2], Guang-yao Yang[4], Qing-pei Yang[4, *]

[1] Ministry of Education Key Laboratory for Earth System Modeling, Center for Earth System Science, Tsinghua University, Beijing 10084, China

[2] The Joint Center for Global Change Studies, Beijing 100875, China

[3] Key Laboratory of Forest Ecology and Management, Institute of Applied Ecology, Chinese Academy of Sciences, Shenyang 110016, China

[4] Jiangxi Provincial Key Laboratory for Bamboo Germplasm Resources and Utilization, Jiangxi Agricultural University, Nanchang 330045, China

* Corresponding author: H Lu

Tel: +86-10-62772565

Fax: +86-10-62797284

E-mail: luhui@tsinghua.edu.cn

Address: Rm. 821, Mengmingwei Sci & Tech Building, Tsinghua University, Haidian District, Beijing 100084, People's Republic of China

* Corresponding author: Q.-P. Yang

Tel: +86-0791-83828029, +86 13479161643

Fax: +86 0791 83828029

E-mail: Qingpeiyang@126.com

Address: No. 1101, Zhimin Road, Changbei Economic Development District, Nanchang 330045, People's Republic of China

Supplementary

Species	Organs	Regression models
Phyllostachys pubescens	Clum	$W_C = 0.231\times DBH^{1.985} \times H^{-0.207}$
	Branch	$W_B = 0.215 \times DBH^{1.303} \times H^{-0.185}$
	Leaf	$W_L = 0.050 \times DBH^{1.695} \times H^{-0.184}$
	Coarse Root	$W_C = 0.008 \times DBH^{0.890} \times H^{1.364}$
		$1/H = 0.047 + 0.276/DBH$
Castanopsis fargesii	Stem	$W_S = 0.112 \times (DBH^2 \times H)^{0.813}$
	Branch	$W_B = 0.001 \times (DBH^2 \times H)^{1.178}$
	Leaf	$W_L = 0.002 \times (DBH^2 \times H)^{0.815}$
	Coarse Root	$W_C = 0.026 \times (DBH^2 \times H)^{0.852}$
		$1/H = 0.016 + 1.149/DBH$
Other trees	Clum	$W_S = 0.017 \times (DBH^2 \times H)1.081$
	Branch	$W_B = 0.021 \times (DBH^2 \times H)^{0.824}$
	Leaf	$W_L = 0.005 \times (DBH^2 \times H)^{0.950}$
	Coarse Root	$W_C = 0.018 \times (DBH^2 \times H)^{0.912}$
		$1/H = 0.016 + 1.149/DBH$

Table 1s Regression models for the biomass of *Phyllostachys pubescens* and trees in Dagangshan National Forest Ecological Station, Jiangxi Province, China. The data are from Yang *et al.*[21]. W_S, stem biomass; W_B, branch biomass; W_L, leaf biomass; W_C, coarse root biomass; DBH, the diameter at breast height. H, the species height.
